# Gene expression in circulating tumor cells reveals a dynamic role of EMT and *PD-L1* during osimertinib treatment in NSCLC patients

**DOI:** 10.1038/s41598-021-82068-9

**Published:** 2021-01-27

**Authors:** Aliki Ntzifa, Areti Strati, Galatea Kallergi, Athanasios Kotsakis, Vassilis Georgoulias, Evi Lianidou

**Affiliations:** 1 Analysis of Circulating Tumor Cells Lab, Lab of Analytical Chemistry, Department of Chemistry, National and Kapodistrian University of Athens, 15771 Athens, Greece; 2 Division of Genetics, Cell and Developmental Biology, Department of Biology, University of Patras, Patras, Greece; 3 Department of Medical Oncology, General University Hospital of Larissa, Larissa, Greece; 4 Hellenic Oncology Research Group (HORG), Athens, Greece

**Keywords:** Non-small-cell lung cancer, Gene expression

## Abstract

Liquid biopsy is a tool to unveil resistance mechanisms in NSCLC. We studied changes in gene expression in CTC-enriched fractions of EGFR-mutant NSCLC patients under osimertinib. Peripheral blood from 30 NSCLC patients before, after 1 cycle of osimertinib and at progression of disease (PD) was analyzed by size-based CTC enrichment combined with RT-qPCR for gene expression of epithelial (*CK-8*, *CK-18*, *CK-19)*, mesenchymal/EMT (*VIM*, *TWIST-1, AXL)*, stem cell (*ALDH-1)* markers, *PD-L1* and *PIM-1.* CTCs were also analyzed by triple immunofluorescence for 45 identical blood samples. Epithelial and stem cell profile (*p* = 0.043) and mesenchymal/EMT and stem cell profile (*p* = 0.014) at PD were correlated. There was a strong positive correlation of *VIM* expression with *PIM-1* expression at baseline and increased *PD-L1* expression levels at PD. *AXL* overexpression varied among patients and high levels of *PIM-1* transcripts were detected. *PD-L1* expression was significantly increased at PD compared to baseline (*p* = 0.016). The high prevalence of *VIM* positive CTCs suggest a dynamic role of EMT during osimertinib treatment, while increased expression of *PD-L1* at PD suggests a theoretical background for immunotherapy in EGFR-mutant NSCLC patients that develop resistance to osimertinib. This observation merits to be further evaluated in a prospective immunotherapy trial.

## Introduction

Over the past two decades, great advances have been made in the therapeutic management of non-small cell lung cancer (NSCLC) patients with somatic mutations in the tyrosine kinase (TK) domain of epidermal growth factor receptor (EGFR). First (gefitinib and erlotinib) and second (afatinib) generation EGFR TKIs have effectively replaced chemotherapy as first line treatment^1^. However, despite initial responses, almost 60% of patients will experience disease progression mainly due to the acquired exon 20 EGFR T790M mutation^2^. Osimertinib, a third generation EGFR TKI, was effectively used as a second line treatment to overcome acquired resistance^3^ and recently was successfully introduced in the first line setting for the untreated EGFR mutant NSCLC patients^4^.

The major challenge that clinicians often face during treatment of NSCLC patients is the heterogeneous landscape of the disease. EGFR reactivations through the presence of tertiary mutations, such as the most frequent exon 20 EGFR C797S mutation, often occur^5^. EGFR-independent mechanisms include the activation of alternative signaling pathways such as MET or HER2 amplification, *PIK3CA* mutations, histological transformation to SCLC and epithelial-to-mesenchymal transition (EMT)^6,7^. Therefore, there is an unmet need to identify biomarkers related to osimertinib resistance, and through proper validation to lead to efficient targeted therapies.

A growing body of evidence reveals the key role of EMT in NSCLC and its involvement in EGFR TKI resistance^7^. The study of the receptor tyrosine kinase AXL (from the Greek word ‘anexelekto’), which has been implicated in EMT, cell survival, invasion, metastasis and drug resistance in several cancers, has also led to a rapidly evolving interest in NSCLC^8^. Several studies suggest that AXL inhibition could confer potent results in the treatment of EGFR mutant NSCLC patients^9–11^. Recent studies indicated the prognostic role of AXL in NSCLC suggesting a new additional tool to customized therapy of EGFR treated patients^12^ and also to the stratification of operable early stage lung adenocarcinoma patients that might benefit from new targeted adjuvant therapy^13^.

Moreover, many studies focus to unravelling the implication of other signaling pathways in NSCLC, and through this information novel potential biomarkers and therapeutic targets arise. Proviral integration site for Moloney murine leukemia virus-1 (PIM-1) is a kinase that is implicated in the control of cancer cell proliferation, migration and apoptosis, by interacting with several other oncogenic signaling pathways and its oncogenic and prognostic role is already proven in various types of cancer including lung cancer^14^. Several studies have shown that PIM-1 indirectly affects EGFR signaling and that its inhibition synergistically improved the efficacy of other inhibitors and subsequently patients’ treatment outcomes^14,15^.

In recent years, immune checkpoint inhibitors (ICI) based on blocking the PD-1/PD-L1 axis have become an important tool for treating advanced NSCLC in first- or second-line setting. However, despite several efforts to assess the efficacy of combining ICI with EGFR TKIs, the role of PD-L1 in EGFR mutant NSCLC and whether EGFR TKI treated NSCLC patients could benefit from combinational or subsequent immunotherapy is still controversial^16^. Interestingly, recent studies have linked resistance to EGFR TKIs with upregulation of PD-L1 in NSCLC patients highlighting a possible benefit from ICI treatment^17,18^.

During the last years, liquid biopsy analysis based mainly on CTCs and circulating tumor DNA (ctDNA) offers a great advantage of minimally invasive monitoring of disease and treatment outcome over time^19–22^. Detection of EGFR mutations in plasma ctDNA of NSCLC patients has successfully paved the road towards personalized medicine always complementary to tissue biopsy^23^. CTCs serve as an alternative biological information source, beyond ctDNA analysis, offering a great potential for unveiling the tumor profile and resistance mechanisms in NSCLC^24–26^. The presence of CTCs in advanced NSCLC, based on CellSearch analyses, constitutes an independent prognostic factor^27^, irrelevant to received therapy^28^. More precisely, in NSCLC patients treated with osimertinib, CTCS were also associated with poorer PFS^29^. The assessment of druggable alterations in CTCs underlie the clinical utility of CTCs to identify therapeutic resistance mutations^30^. Additionally, the presence of distinct CTC subpopulations could be predictive for different treatment outcomes^31,32^. NSCLC patients have also benefited from CTC molecular characterization including the detection of *PD-L1* expression^33–35^.

The objective of this study was to explore changes in gene expression in CTCs of EGFR-mutant NSCLC patients under osimertinib treatment. To achieve this, we performed an RNA-based molecular characterization of CTCs for the same patients before, during and after osimertinib treatment, and followed changes in the mRNA expression of epithelial, mesenchymal/EMT and stem cell markers as well as potential therapeutic targets like *PD-L1, PIM-1* and *AXL*. To the best of our knowledge, this is the first study on gene expression in CTCs at three different time points in patients under osimertinib treatment.

## Results

**Figure 1:**
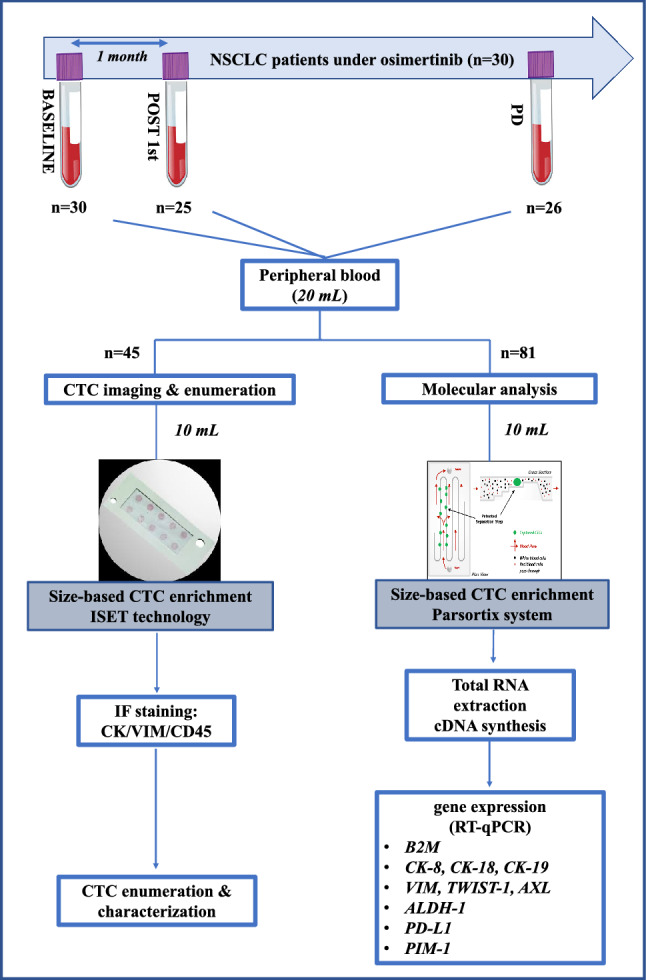
A schematic flowchart of the study.

The outline of the study is shown in Fig. 1.

### Gene expression profile of size-based enriched CTC-fractions

**Figure 2:**
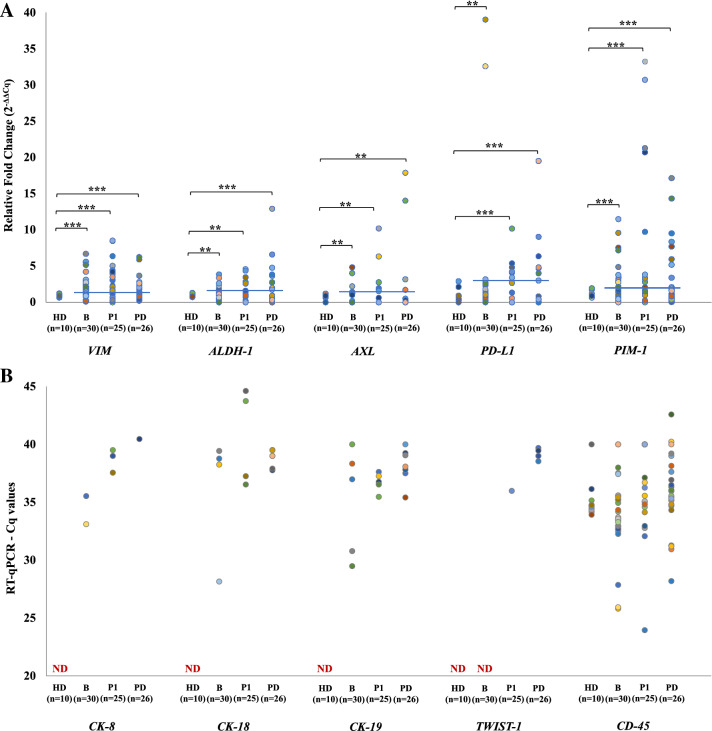
Gene expression levels for size-based CTC-enriched fractions for NSCLC patient samples at baseline (n = 30), at post-1st cycle (n = 25) and at PD (n = 26) and for HD (*n* = 10): (**A**) relative fold change values (2^–ΔΔCq^) in respect to *B2M* expression for: *VIM*, *ALDH-1, AXL*, *PD-L1*, and *PIM-1,* (**B**) Cq values for *CK-8, CK-18, CK-19, TWIST-1,* and *CD45* (ND: non detected). ***P < 0.001, **P < 0.05.

Spiking experiments using a known number of NCI-H1975 (10, 100, 1000 cells) spiked in 10 mL peripheral blood (PB) of healthy donors (HD) have shown that following Parsotrix enrichment, these cells were detected through *CK-19* mRNA expression in all cases (Supplementary Fig. S1). To evaluate RT-qPCR specificity for each gene, we analyzed in exactly the same way peripheral blood samples from 10 HD. In the HD group *CK-8, CK-18, CK-19* and *TWIST-1* transcripts were not detected in any sample, while *VIM*, *ALDH-1*, *AXL, PD-L1* and *PIM-1* transcripts were detected at low levels (Fig. 2). Thus, in all patient samples RT-qPCR data for these genes were normalized in respect to the expression of *B2M* reference gene by using the 2^–ΔΔCq^ approach, as previously described^36^. The cut-off values for *VIM*, *ALDH-1*, *AXL, PD-L1* and *PIM-1* transcripts were calculated as the mean of signals derived in the HD group, analyzed in exactly the same way, plus 2SD (Supplementary Table S1) as previously described^37–39^. The absolute number of *VIM*, *ALDH-1*, *AXL, PD-L1* and *PIM-1* transcripts was significantly higher in patients’ samples in comparison to HD, at all time points (Fig. 2A). *CK-8, CK-18, CK-19* and *TWIST-1* transcripts were detected in patient samples at all time points, but their values were not normalized since they are not expressed at all in healthy donors (Fig. 2B). We have also examined the expression levels of *CD45* (general leukocyte marker) in CTC-enriched fractions isolated through Parsotrix system. Expression of *CD45* in the CTC-enriched fractions indicated a contamination of leucocytes that are expected to be co-isolated (Fig. 2B).

**Figure 3:**
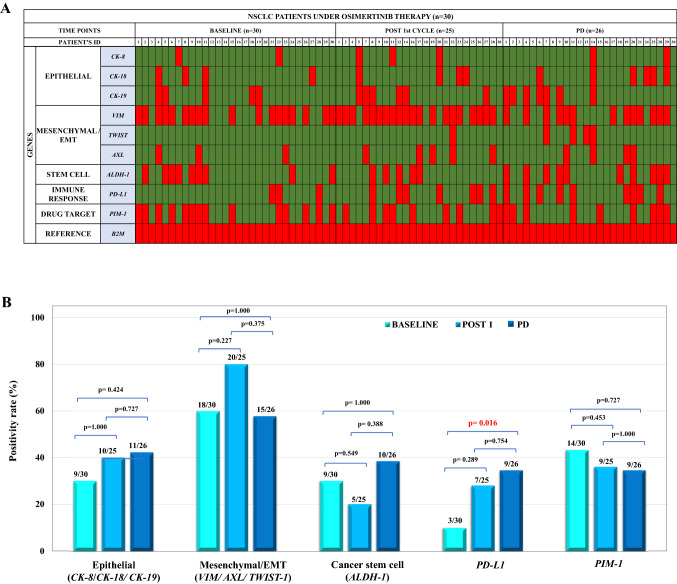
(**A**) Gene expression profile of CTCs: (a) before treatment with osimertinib (n = 30) (b) after one cycle of treatment (n = 25) and (c) at progression of disease (PD) (n = 26) (red: overexpressed, green: no expression). SD: stable disease, PR: partial response, CR: complete response, PD: progression of disease. (**B**) Positivity rates of gene expression in CTCs at all time points. Expression of epithelial, mesenchymal/EMT, stem cell markers, *PD-L1* and *PIM-1*, at baseline, post-1st cycle, and PD.

The heat map shown in Fig. 3A, is summarizing all data, and demonstrates a significant heterogeneity on gene expression in CTC-enriched fractions among NSCLC patients at all time points [before treatment, post-1st cycle, and progression of disease (PD)]. The expression of epithelial markers (at least one; *CK-8*, and/or *CK-18,* and/or *CK-19)* was detected in 30 out of 81 (37%) samples, the expression of mesenchymal/EMT markers (at least one; *VIM,* and/or *TWIST-1,* and/or *AXL*) in 53 out of 81 (65.4%) and the expression of the stem cell marker *ALDH-1* in 24 out of 81 (29.6%). Differences within every time point and differences between time points for the samples analyzed were also evaluated. Τhe gene expression patterns during all time points are described in detail below:

#### Epithelial markers

Before treatment, in 9 out of 30 (30%) patients at least one epithelial marker was detected; *CK-8* transcripts were detected in 2 out of 30 (6.67%), *CK-18* transcripts were detected in 4 out of 30 (13.3%), *CK-19* in 5 out of 30 (16.7%) baseline samples. After one cycle of treatment, at least one epithelial marker was detected in 10 out of 25 (40%) patients; *CK-8* transcripts were detected in 3 out of 25 (12%), *CK-18* transcripts were detected in 4 out of 25 (16%), and *CK-19* in 6 out of 25 (24%) samples. At progression of disease, in 11 out of 26 (42.3%) patients epithelial markers in CTC-enriched fractions were detected; *CK-8* transcripts were detected in 2 out of 26 (7.69%) samples, *CK-18* transcripts were detected in 6 out of 26 (23.1%) and *CK-19* in 7 out of 26 (26.9%). Although the expression of epithelial markers was increased at PD compared to that of baseline samples there was no significant difference between the two time points (McNemar test, *p* = 0.424) (Fig. 3B, Table 1).

**Table 1:** Comparison of gene expression in CTC-enriched fractions before treatment with AZD9291 and at progression of disease (PD).

Gene target		PD				
	Baseline	* − *	+	Total	Concordance	McNemar test, *p*
Epithelial *(CK-8, CK-18, CK-19)*	* − *	10	9	19		
	+	5	2	7	12/26 (46.1%)	0.424
	Total	15	11	26		
Mesenchymal/EMT *(VIM, TWIST-1, AXL)*	−	4	7	11		
	+	7	8	15	12/26 (46.1%)	1.000
	Total	11	15	26		
Stem cell *(ALDH-1)*	−	10	7	17		
	+	6	3	9	13/26 (50%)	1.000
	Total	16	10	26		
*PD-L1*	−	17	7	24		
	+	0	2	2	19/26 (73.1%)	0.016
	Total	17	9	26		
*PIM-1*	* − *	12	3	15		
	+	5	6	11	18/26 (69.2%)	0.727
	Total	17	9	26		

#### Mesenchymal/EMT markers

Before treatment, in 18 out of 30 (60%) patients at least one mesenchymal/EMT marker was detected; *VIM* transcripts were detected in 18 out of 30 (60%), *AXL* overexpression was detected in 3 out of 30 (10%), whereas *TWIST-1* transcripts were not detected at all (0%). After one cycle of treatment, in 20 out of 25 (80%) patients at least one mesenchymal/EMT marker was detected; *VIM* transcripts were detected in 20 out of 25 (80%), *AXL* in 5 out of 25 (20%), whereas *TWIST-1* was detected in 1 out of 25 (4%). At progression of disease, in 15 out of 26 (57.7%) patients at least one mesenchymal/EMT marker was detected; *VIM* transcripts were detected in 13 out of 26 (50%), *AXL* transcripts were detected in 4 out of 26 (15.4%) and *TWIST-1* in 4 out of 26 (15.4%) samples. No significant differences were observed for mesenchymal/EMT markers in CTC-enriched fractions detected at baseline, post-1st cycle and PD (McNemar test, *p* = 1.000; Fig. 3B, Table 1).

#### *ALDH-1*

Before treatment, *ALDH-1* transcripts were detected in 9 out of 30 (30%) samples (Fig. 2B), after one cycle in 5 out of 25 (20%) and at progression of disease in 10 out of 26 (38.5%). No significant differences were observed for *ALDH-1* in CTC-enriched fractions detected at baseline post-1st cycle and at PD (McNemar test, *p* = 1.000; Fig. 3B, Table 1).

At baseline, a significant correlation was observed between the expression of mesenchymal/ EMT markers and *ALDH-1* (Fisher’s exact test, *p* = 0.049). No other significant correlation was found between epithelial and mesenchymal or *ALDH-1* (Fisher’s exact test, *p* = 0.084, and *p* = 0.071, respectively). More precisely, in 6/30 (20%) cases, epithelial markers and *VIM* were co-expressed in the CTC-enriched fractions of these patients (Supplementary Table S2). After one cycle of treatment, no significant correlations were observed between the three CTC subtypes in this group of samples when compared in pairs [Fisher’s exact test, epithelial-mesenchymal (*p* = 0.312), epithelial- *ALDH-1* (*p* = 1.000), mesenchymal- *ALDH-1* (*p* = 0.544)] (Fig. 3B). The expression of all three CTC subtypes was no different after one cycle of treatment [McNemar test, epithelial (*p* = 1.000), mesenchymal (*p* = 0.227) or *ALDH-1* (*p* = 0.549)] compared to that at baseline. At PD, by comparing the different CTC profiles, significant correlations between epithelial profile and *ALDH-1* (Fisher’s exact test, *p* = 0.043) and mesenchymal/EMT and *ALDH-1* (Fisher’s exact test, *p* = 0.014) were observed. In 7/26 (26.9%) cases, epithelial markers and *VIM* were co-expressed in the CTC-enriched fractions of these patients. In 20/81 (24.7%) of the total number of cases studied, epithelial markers and *VIM* were co-expressed in the CTC-enriched fractions of these patients (Supplementary Table S2).

#### *PD-L1*

*PD-L1* expression was low at baseline (3 out of 30 patients; 10%) but tended to increase after the first cycle of treatment [7 out of 25 patients (28%); McNemar test, *p* = 0.289]. Interestingly, there was a significant difference in *PD-L1* expression levels between baseline and disease progression (9 out of 26 (34.6%), (McNemar test, *p* = 0.016; Fig. 3B, Table1). At baseline and at post-1st treatment cycle there was no positive correlation between *PD-L1* expression and any of the markers of the three different CTC subtypes. Interestingly, at PD, a correlation was found between *PD-L1* status and the mesenchymal subtype (Fisher’s exact test, *p* = 0.036) and more precisely with *VIM* overexpression (Fisher’s exact test, *p* = 0.011). No correlation was observed between *PD-L1* and *ALDH-1* expression at PD (Fisher’s exact test, *p* = 0.234).

#### *PIM-1*

*PIM-1* overexpression was detected in 14 out of 30 (46.7%) baseline samples, in 9 out of 25 (36%) at post-1st cycle samples and in 9 out of 26 (34.6%) at disease progression but there was no statistically significant difference among these time points (Fig. 3B). At baseline, a stronger positive correlation was observed between *PIM-1* overexpression and mesenchymal/EMT profile and more precisely with VIM expression (Fisher’s exact test, *p* = 0.001) compared to that for the post-1st cycle samples (Fisher’s exact test, *p* = 0.621) and for the PD samples (Fisher’s exact test, *p* = 0.217). In respect to correlations between *PIM-1* and epithelial profile there was no correlation at any of the three time points (baseline, post-1st cycle and PD: Fisher’s exact test, *p* = 0.399, *p* = 0.401, and *p* = 0.103, respectively). In addition, a moderate correlation was observed for *ALDH-1* (Fisher’s exact test, *p* = 0.046) at PD whereas no correlation was observed at baseline (Fisher’s exact test, *p* = 0.236) and at post-1st cycle samples (Fisher’s exact test, *p* = 0.312). *PIM-1* expression was similarly high at all time points and there was no significant difference between baseline and PD (McNemar test, *p* = 0.727; Table 1).

### Detection of ISET-enriched CTCs by triple IF

**Figure 4:**
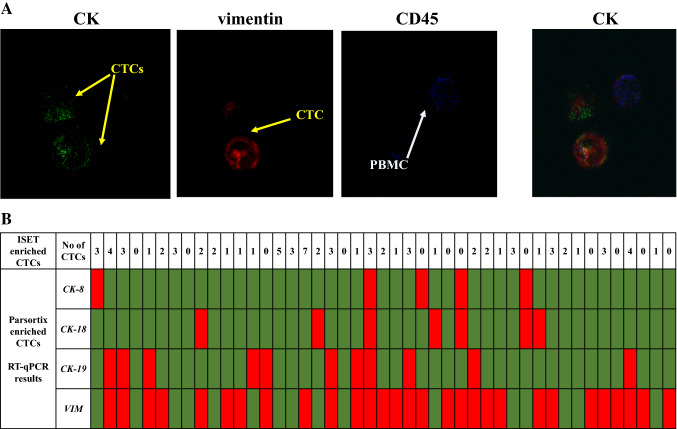
(**A**) Representative IF images of ISET-enriched CTC: Vimentin positive and negative CTCs. Cells were stained with CK (green), Vimentin (red) and CD45 (blue), Dapi (not shown). CTCs phenotypes in NSCLC patients were identified using Confocal laser scanning microscopy (magnification X40), (**B**) Direct comparison of size-based enriched CTC-fractions using molecular analysis at the gene expression level (Parsortix) and IF analysis (ISET), using the same blood draws (n = 45).

Using identical blood draws from the same group of patients (n = 30), an additional PB sample (10 mL in EDTA) was available from 45 matched samples from different time points for CTC isolation using the ISET technology. A representative image from CTCs isolated with the ISET is shown in Fig. 4A. Triple immunofluorescence analysis has shown that 34 out of 45 (75.6%) samples were positive for *CK* (pan cytokeratin Ab, CK-8, CK-18, CK-19) and/or *VIM* (Fig. 4B). Twenty five samples were found positive and three samples were found negative by both RT-qPCR and triple IF (Fig. 4B). However, as can be seen in Table 2, there was a balanced discrepancy for 17 samples. The comparison of the triple IF results to RT-qPCR for mesenchymal/EMT CTC subpopulation detection, did not reveal any significant difference (McNemar test, *p* = 1.000) (Table 2).

**Table 2:** Comparison of CTC detection using RT-qPCR (CTC size-based enrichment with Parsortix) based on the expression of *CK-8, CK-18,* and/or *CK-19,* and/or *VIM* and IF staining (CTC size-based enrichment with ISET) based on CK-8/CK-18/CK-19, and/or VIM detection (n = 45).

		IF CK + /VIM + /CD45- and/or CK + /VIM-/CD45-		
	RT-qPCR	−	+	Total
*CK-8* (+), *CK-18* (+), *CK-19* (+), *VIM* (+)	−	3	9	12
	+	8	25	33
	Total	11	34	45

Concordance: 28/45 (62.2%).

McNemar test, *p* = 1.000.

## Discussion

We report a high heterogeneity in gene expression of CTC-enriched fractions among NSCLC patients during osimertinib therapy and also between the different time points for each patient. Our results on the gene expression profile of size-based enriched circulating tumor cells reveal a dynamic role of EMT and PD-L1 during osimertinib treatment in EGFR-mutant NSCLC patients. To the best of our knowledge, this is the first study on gene expression in CTCs at three different time points in patients under osimertinib treatment.

Molecular characterization of CTCs at the gene expression level has a strong potential to provide information on tumor heterogeneity and unravel oncogenic alterations related to metastasis or to treatment sensitivity and resistance^20,22^. Our results indicate that the expression of epithelial markers increased at PD compared to baseline but without any statistically significant difference. Despite the heterogeneous epithelial profile among patients, osimertinib seems to have no effect on the expression of epithelial markers in CTCs, and the expression of mesenchymal and stem cell markers showed no differences in total among all time points.

Interestingly, in the total number of samples analyzed, CTCs expressed the highest levels of the mesenchymal/EMT profile (65.4%). More precisely, *VIM* expression was observed in high rates at all time points of therapy, indicating a role of EMT in these CTCs during osimertinib treatment. Several studies demonstrated the upregulation of vimentin in EGFR TKI resistant cells and pointed out that EMT might be one of possible mechanisms for acquired resistance to EGFR TKIs^40–42^. Additionally, despite the low rates of *TWIST-1* overexpression observed in our samples, according to previous evidence targeting TWIST-1 may be an option to overcome mediated resistance to osimertinib^43^. Therefore, targeting EMT regulators could be an alternative therapeutic approach in EGFR mutant NSCLC^40,43^.

The high percentage of mesenchymal/EMT profile markers could be explained by the biomarker-independent CTC enrichment methodology used, which also assures the efficient depletion of contaminating leukocytes, thus providing a high purity of the enriched CTCs^44^. Our data on *CD45* expression in CTC-enriched CTC fractions clearly indicate that by using a size-exclusion and biomarker independent CTC enrichment methodology we do co-isolate a small amount of leucocytes as expected, but this amount is relatively small, and does not affect our results since we normalize our data for genes that are co-expressed in CTC and PBMC using the expression levels of the healthy donor group samples that were analyzed in exactly the same way.

Previous direct comparisons of CellSearch system with size-based enrichment technologies revealed higher frequencies of CTCs in advanced NSCLC while using the latter EpCAM independent method^45,46^. Moreover, epithelial independent enrichment methods unveiled the presence of different CTC subpopulations, other than EpCAM positive CTCs, that exhibit a more aggressive or stem-like character especially in EGFR altered CTCs^31,46^. Vimentin positive CTCs in advanced NSCLC patients are of high abundance indicating the involvement of EMT in drug resistance^30,46^. According to a very recent study, based on a similar size-based CTC enrichment methodology, the majority of recovered CTCs/clusters were EpCAM-negative, suggesting that these cells would have been missed using traditional antibody-based capture methods^47^.

The presence of CTCs in the PB of the studied cohort of patients, detected after Parsortix enrichment, was in parallel verified by using a combination of the size-based isolation platform, ISET and confocal microscopy in 45 matched samples from all time points. A direct comparison, in these samples, analyzed by RT-qPCR for cytokeratin and vimentin in enriched CTCs, revealed an agreement on CTC positivity between the two platforms. Overall, we could efficiently enrich CTCs by using either Parsortix or ISET and further detect and characterize them in combination with RT-qPCR or IF staining, respectively.

We also report a positive correlation between the mesenchymal/EMT and stem cell markers at baseline and at PD that might be representative of the molecular association between EMT and stemness. It is demonstrated that there is molecular link between the activation of the EMT and a CSC state, transfusing a more malignant phenotype in cancer cells^48^. Another important aspect of this EMT regulation is the implication of EMT induced CSC in therapeutic resistance^48^. Moreover, the presence of CSC due to EGFR inhibition has already been shown in EGFR mutated NSCLC cell lines and was correlated with acquired resistance to osimertinib^49^.

In our study, we evaluated for the first time the mRNA expression levels of the EMT-connected *AXL* gene in CTCs of NSCLC during osimertinib therapy. Our experiments have shown increased levels of *AXL* transcripts in CTCs after the first cycle of treatment and at PD in comparison to baseline. These results are consistent with a previous study which underlines the presence of *AXL* either in the initial phase or tolerant phase of treatment with osimertinib indicating that a group of patients overexpressing *AXL* may synergistically benefit from AXL inhibitors^11^. In a recent study, *AXL* expression in CTCs was mostly detected in *VIM-*positive CTCs indicating a role of *AXL* in EMT^50^. Zhang et al.^51^ have also shown a possible EMT role of *AXL* in the development of acquired EGFR TKI resistance, which is marked by vimentin overexpression. In our study, *AXL* mRNA expression was not significantly correlated with *VIM* mRNA expression in CTCs, however, 10/11 (90.9%) of *AXL* positive samples were also positive for *VIM*. This could possibly be explained by the fact that CTCs are highly heterogeneous and our results were based on bulk analysis of CTCS, while in the above referenced studies AXL expression was correlated with vimentin in the primary tumors^50,51^.

We also examined *PIM-1* expression in CTCs in this patient cohort since it is a promising novel therapeutic target in NSCLC. Many studies indicate the synergistic effects of combination of PIM inhibitor and osimertinib either by preventing the activation of oncogenic signaling pathways^15^ or acting through the inhibition of the phosphorylation of STAT3^52^. Herein, we report that relatively high levels of *PIM-1* transcripts were detected in all time points of our study, without any statistically significant difference among them; this observation strongly suggests that this kinase in constantly highly expressed in CTCs and, in parallel, its expression is not affected by osimertinib. However, a strong positive correlation was found between *PIM-1* and *VIM* expression mostly at baseline in contrast to PD samples; although, there is no a clear explanation for this finding, it could be speculated that, according to previous evidence, PIM-1 might indirectly promote cell proliferation by regulating signaling pathways such as IL-6/STAT3^15,53^.

Recent studies in EGFR-driven NSCLC specimens indicate that the EGFR signaling pathway plays an important though controversial role in regulating *PD-L1* expression in human NSCLC cells^54^. Preclinical evidence demonstrated that continuous exposure to EGFR TKIs induces PD-L1 expression in resistant NSCLC^17^. This was also verified by studies in patient cohorts in which higher levels of PD-L1 expression at disease progression during EGFR TKI treatment were described^18,55^. Such changes in the tumor microenvironment (TME) as a result of different resistance mechanisms^55^ combined with their favorable PFS and OS outcomes^18^ might represent a possibility for subsequent ICI treatment. On the other hand, few recent studies have shown that osimertinib caused down-regulation of *PD-L1* mRNA expression in EGFR mutant NSCLC cell lines^56,57^. Our results revealed that *PD-L1* expression levels in CTCs tended to significantly increase at disease progression after osimertinib treatment (34.6%) and they are in accordance with previous findings that attribute upregulation of PD-L1 at resistance to EGFR TKIs implicating that a subgroup of patients could benefit from ICI treatment following EGFR targeted therapy^17,18^. Another interesting finding was the significant correlation between *PD-L1* status and *VIM* overexpression, consistent with previous studies that associate EMT with activation of TME^58^.

Our results demonstrated the heterogeneous patterns of gene expression of epithelial, mesenchymal/EMT and stem cell markers among patients. The EpCAM-independent CTC enrichment approach permitted the detection of vimentin positive CTCs at high rates at all time points indicating a potential role of EMT during osimertinib treatment. Our observations could support further studies, including larger cohorts of patients, to clarify the potential role of *PIM-1* and *AXL* as novel CTC biomarkers and therapeutic targets in NSCLC. The significant increase in the expression levels of the immune response marker *PD-L1* in CTCs at disease progression suggests a theoretical background for immunotherapy in EGFR-mutant NSCLC patients that develop resistance to osimertinib. This observation merits to be further evaluated in a prospective immunotherapy trial.

## Materials and methods

### Patients

Patients with histologically or cytologically documented EGFR mutated lung adenocarcinomas were treated with second line osimertinib (AZD9291; Astra Zeneca, UK) in the context of a multicenter Phase II clinical study [ClinicalTrials.gov number: NCT02771314, registration date: 13/05/16 and EudraCT number: 2016-001335-12, registration date: 13/04/16] conducted by the Hellenic Oncology Research Group (HORG). The study was conducted in accordance with the Declaration of Helsinki and has been approved by the National Drug Administration (EOF), the National Ethics Committee (35/00-03/16, 35/03-11/16) and the Institutional Ethical Committees of the HORG’s participating centers. All patients gave their written informed consent to participate. In total 48 enrolled patients were enrolled in the clinical trial, but only for 30 of those peripheral blood samples were available for CTC analysis and thus were included in the current study (Supplementary Fig. S2). We present here a study on gene expression analysis in CTC-enriched fractions in a subgroup of these patients at three time points: (a) baseline, 30 patients, (b) post 1st line treatment, 25/30 patients, and (c) progression of disease (PD) 26/30 (these patients had experienced disease progression while 4 out of 30 are still under therapy. The median age of patients was 67.5 (range: 43–87 years) and 19 (63.3%) of them were female. 23 (76.7%) and 7 (23.3%) patients were previously treated with 1st and 2nd generation EGFR TKIs, respectively. Osimertinib was administered as a 2nd line treatment in 15 (50%) of them and as 3rd line treatment or further in the others (Supplementary Table S3). All patients had a documented disease progression upon 1st and/or 2nd generation EGFR TKIs, a Performance Status (ECOG) 0–1 and gave their written informed consent to participate in the study which has been approved by the National Drug Administration (EOF), the National Ethics Committee as well as by the Institutional Ethical Committees of the HORG’s participating centers. Peripheral blood (PΒ) was obtained for the patients included in this study (n = 30) at three time points: (a) before the treatment initiation with osimertinib (baseline: n = 30 samples); (b) after one cycle of treatment (post-1st cycle: n = 25 samples) and (c) at the time of disease progression (PD: n = 26 samples). In total, 81 patient samples from different time points were analyzed for gene expression in CTCs. In addition, 10 HD were used as a control group. Patients’ and HD’s PB was obtained and analyzed in exactly the same way.

### CTC enrichment for molecular analysis

PB (10 mL) was collected in EDTA tubes, after discarding the first 5 mL of blood draw to avoid contamination of skin epithelial cells. Blood samples were centrifuged at 530 ×g for 10 min at room temperature (RT) and plasma was removed and kept at − 70 °C for further analysis. Equal volume of removed plasma was replaced by adding phosphate buffered saline (PBS, pH 7.3) into the cell pellet and then samples were proceeded for CTC enrichment in the size-based microfluidic device, Parsortix (ANGLE plc, UK) using a cassette with a 6.5 μm separation. The harvested cells were collected in a final volume of 210 μL PBS. The human lung cancer cell line NCI-H1975 was used to evaluate the recovery ratio for CTC enrichment. For this reason, serial dilutions of known numbers of NCI-H1975 cells (10, 100, 1000 cells) were prepared and spiked into 10 mL PB of HD and then enriched by Parsortix. All spiked samples were further analyzed for *CK-19* expression by RT-qPCR^59^. Exactly the same cell preparations of NCI-H1975 cells that were not subjected to spiking and enrichment were also analyzed for *CK-19* expression to assess the recovery rates.

### RNA isolation-cDNA synthesis

After CTC enrichment using the Parsotrix platform, total RNA was extracted from the harvested cells, followed by cDNA synthesis, as previously described^36^. TRIZOL LS (Thermo Fisher Scientific, USA) was used for the isolation of total RNA under RNAase free conditions. After isolation, RNA was dissolved in Ambion RNA Storage Solution (Thermo Fisher Scientific, USA) and stored at − 70 °C. RNA concentration was measured in a NanoDrop ND-1000 UV–Vis Spectrophotometer (Thermo Fisher Scientific, USA). cDNA synthesis was performed using the High-capacity RNA-to-cDNA kit (Applied Biosystems, USA) in a total volume of 20 uL, according to the manufacturer’s instructions as previously described^36^.

### RT-qPCR

RT-qPCR was performed to evaluate gene expression for a panel consisting of the following genes: (a) epithelial markers (*CK-8*, *CK-18*, *CK-19*), (b) mesenchymal/EMT markers (*Vimentin*, *TWIST-1, AXL*), (c) stem cell marker (*ALDH-1*), (d) immune response marker *PD-L1* and e) *PIM-1* a potential therapeutic target. *B2M* was used as a reference gene for relative quantification, but also for ensuring the presence of amplifiable material in all samples and to avoid false-negative results, as previously described^39^. RT-qPCR assays for the quantification of *CK-19*, *ALDH-1*, *TWIST-1, PD-L1* and *PIM-1* transcripts were performed as previously reported^37–39,59^. The RT-qPCR assays for the quantification of *CK-8*, *CK-18, VIM* and *AXL* transcripts were de novo designed and analytically validated before use. Initially, in silico primer design for these genes was performed using the Primer Premier 5.0 software (Premier Biosoft, CA). The design of the primers was based on the use of BLAST Sequence Similarity Search tool (NCBI, NIH) in order to completely avoid primer-dimer formation, false priming sites, formation of hairpin structures and hybridization to genomic DNA while amplify specifically only the target genes. Detailed optimization experiments were carried out (results not shown). All RT-qPCR assays were performed in the COBAS z480 system (Roche Molecular Systems, Inc.).

### CTC immunofluorescence analysis (IF)

Using identical blood draws from the same group of patients (n = 30), an additional PB sample (10 mL in EDTA) was available from 45 matched samples from different time points for CTC isolation using the ISET technology (Rarecells Diagnostics, France). CTCs were captured in the ISET filters according to the manufacturer’s instructions and were then triple stained by immunofluorescence for CK/VIM/CD45 according to a validated protocol and analyzed using the Confocal laser Scanning microscopy (LEICA), as previously described^60^. Specifically, for cytokeratins (CK) staining, two different antibodies were used as a cocktail: the A45-B/B3 anti-mouse Ab recognizing the CKs 8/18/19 (Micromet Munich, Germany) and an anti-mouse Ab against CK7 (Abcam, Cambridge, UK). Alexa 488 (Invitrogen Carlsbad, CA, USA) anti-mouse was used as a secondary antibody. Anti-CD45 antibody conjugated with Alexa 647 (Novus Biologicals, USA) was also added. Spots were stained with Vimentin antibody (Santa Cruz, Santa Cruz, CA, USA). Finally, slides were stained with DAPI conjugated with antifade (Invitrogen, Carlsbad, CA, USA).

### Statistical analysis

Statistical analysis was performed using IBM SPSS Statistics for Windows, version 25.0 (IBM Corp., Armonk, N.Y., USA). Concordance of expression for each gene between different time points and concordance between RT-qPCR and IF was assessed using the McNemar test. The Pearson chi-square test of independence or Fisher’s exact test were used to make comparisons between groups. The Kolmogorov–Smirnov non-parametric test was used to compare continuous variables between groups. All p-values are two-sided. A level of *p* < 0.05 is considered statistically significant.

## Supplementary Information

Supplementary Information.
