# Supplementary material for: Gene expression in circulating tumor cells reveals a dynamic role of EMT and PD-L1 during osimertinib treatment in NSCLC patients
Source: Sci Rep. 2021 Jan 27;11:2313. doi: 10.1038/s41598-021-82068-9 (PMC7840727; doi:10.1038/s41598-021-82068-9)
Supplement: Supplementary file 1 — Supplementary Information. [file 41598_2021_82068_MOESM1_ESM.pdf]

Gene expression in circulating tumor cells reveals a dynamic role of EMT and *PD-L1* during osimertinib treatment in NSCLC patients

Aliki Ntzifa[1], Areti Strati[1], Galatea Kallergi[2], Athanasios Kotsakis[3], Vassilis Georgoulias[4], Evi Lianidou[1]*

[1]Analysis of Circulating Tumor Cells Lab, Lab of Analytical Chemistry, Department of Chemistry, National and Kapodistrian University of Athens, 15771, Athens, Greece

[2]Division of Genetics, Cell and Developmental Biology, Department of Biology, University of Patras, Patras, Greece

[3]Department of Medical Oncology, University General Hospital of Larisa, Larisa, Greece

[4]Hellenic Oncology Research Group (HORG), Athens, Greece

Correspondence to:

Evi Lianidou, PhD,

Analysis of Circulating Tumor Cells Lab, Lab of Analytical Chemistry,

Department of Chemistry, National and Kapodistrian University of Athens,

15771, Athens, Greece Email: lianidou@chem.uoa.gr

SUPPLEMENTARY FIGURE S1

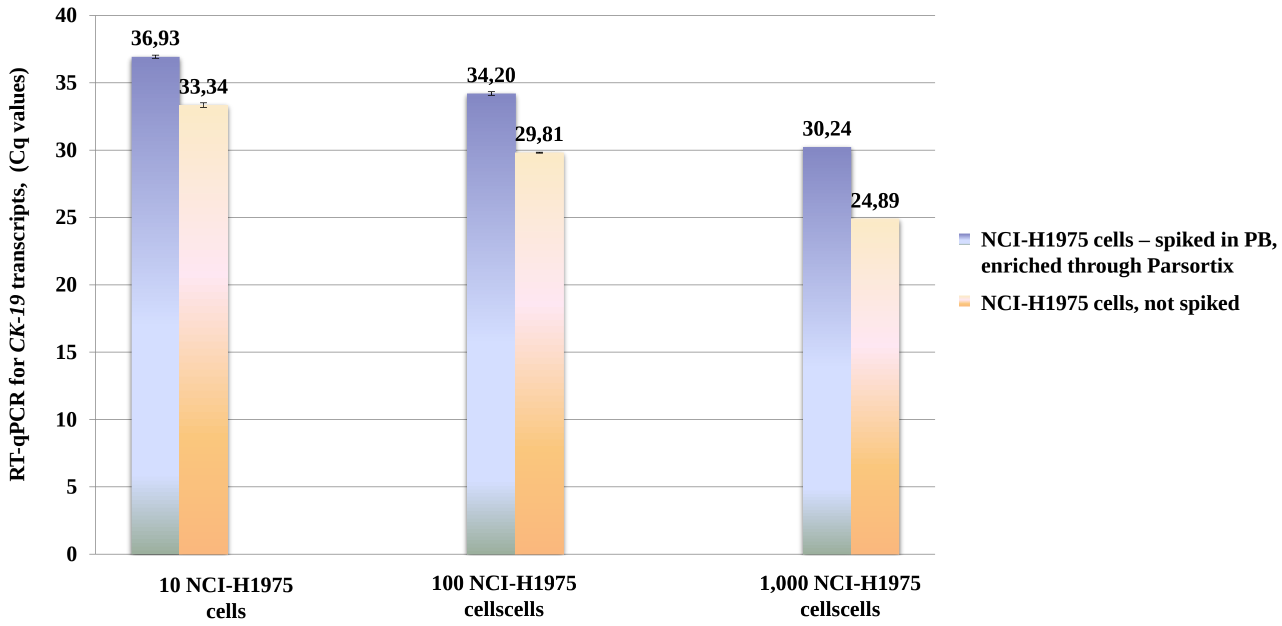

Supplementary Figure S1: Spiking experiments using a known number of NCI-H1975 (10, 100, 1000 cells) spiked in 10mL peripheral blood (PB) of healthy donors (HD) in comparison with NCI-H1975 10, 100, 1000 not spiked cell preparations for *CK-19* mRNA expression.

SUPPLEMENTARY FIGURE S2

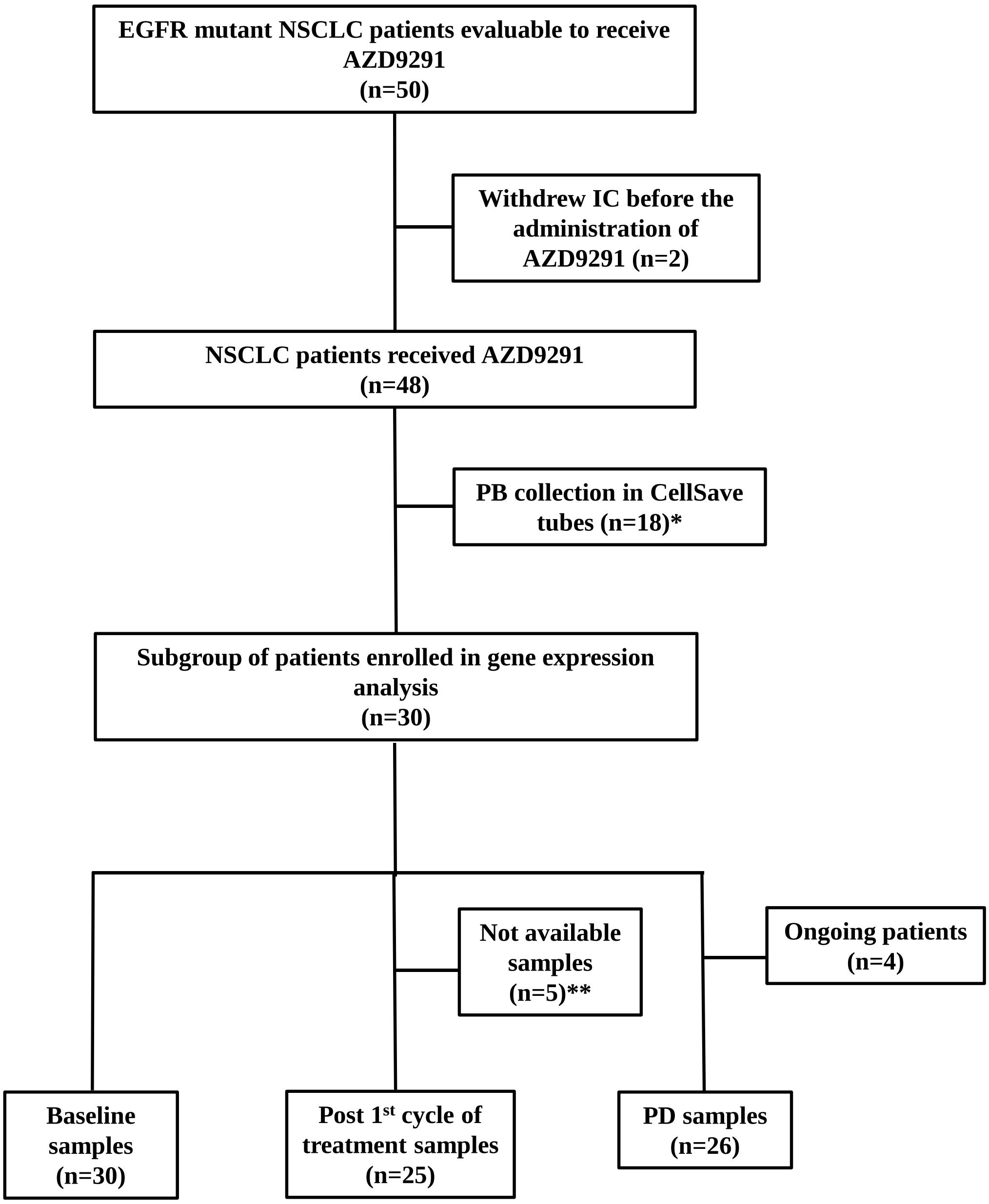

*** CellSave tube's preservatives negatively affect gene expression analysis**

**** not enough PB material (n=3) or not available Post 1st sample because patients progressed within the 1st month of treatment (n=2)**

Supplementary Figure S2: Consort diagram of the study

SUPPLEMENTARY TABLES

Supplementary Table S1: Positivity rates (median fold change values) for the expression of *VIM*, *ALDH-1*, *AXL*, *PD-L1* and *PIM-1* in CTCs at: a) baseline (*n* = 30), b) post 1st treatment (n=25) and c) PD (n=26) in comparison to the control group (HD, *n* = 10).

	**HD**	**Baseline Positivity rate**			**Post 1st treatment Positivity rate**			**Progression of disease (PD) Positivity rate**		
**GENES**	**median fold change (range)**	**no of samples (%)**	**median fold change (range)**	**K-S[a] test (Z, p)**	**no of samples (%)**	**median fold change (range)**	**K-S[a] test (Z, p)**	**no of samples (%)**	**median fold change (range)**	**K-S[a] test (Z, p)**
VIM	0.95 (0.66-1.36)	18/30 (60%)	2.69 (1.35-6.68)	2.214, <0.001	20/25 (80%)	2.68 (1.33-8.50)	2.214, <0.001	13/26 (50%)	2.45 (1.44-6.22)	2.367, <0.01
ALDH-1	1.04 (0.72-1.32)	9/30 (30%)	2.30 (1.00-3.84)	1.829, 0.002	5/25 (20%)	3.11 (1.60-4.56)	1.85, 0.002	10/26 (38.5%)	3.78 (1.77-12.91)	2.108, <0.001
AXL	0.73 (0.0-1.95)	3/30 (10%)	4.00 (2.22-4.82)	1.535, 0.018	5/25 (24%)	2.75 (1.56-10.20)	1.517, 0.02	4/26 (15.4%)	8.60 (1.72-17.88)	1.401, 0.039
PD-L1	0.45 (0.0-2.90)	3/30 (10%)	2.30 (1.00-3.84)	1.519, 0.02	7/25 (28%)	3.11 (1.60-4.56)	2.029, <0.001	9/26 (34.6%)	3.78 (1.77-12.91)	2.176, <0.001
PIM-1	0.89 (0.65-1.95)	14/30 (46.7%)	3.43 (2.10-11.47)	2.207, <0.001	9/25 (36%)	6.76 (2.81-33.24)	2.029, <0.001	9/26 (34.6%)	7.67 (2.10-17.15)	1.984, <0.001

[a]Kolmogorov-Smirnov test (Z, p)

Supplementary Table S2: Co-expression of epithelial markers (*CK-8*, *CK-18*, *CK-19*) and *VIM* in CTCs at different time points.

	**Gene target**		***VIM+***				
			**-**	**+**	**TOTAL**	**CK$^+$VIM$^+$/ total number**	**Fisher's exact test, p**
**BASELINE**	**Epithelial (*CK-8, CK-18, CK-19*)**	**-**	9	12	21	6/30 (20%)	0.704
		**+**	3	6	9		
		**TOTAL**	12	18	30		
POST 1st CYCLE		**-**	2	13	15	7/25 (28%)	0.358
		**+**	3	7	10		
		**TOTAL**	5	20	25		
PD		**-**	9	6	15	7/26 (26.9%)	0.428
		**+**	4	7	11		
		**TOTAL**	13	13	26		
			**-**	**+**	**TOTAL**	**CK$^+$VIM$^+$/ total number**	**Pearson's chi-square, p**
TOTAL (ALL SAMPLES)		**-**	20	31	51	20/81 (24.7%)	0.280
		**+**	10	20	30		
		**TOTAL**	30	51	81		

Supplementary Table S3: Patients' characteristics (n=30)

Characteristics	n (%)
**Age (years)**	
Median	67.5
Range	43-87
Gender	
Male	11/30 (36.7%)
Female	19/30 (63.3%)
**Response**[1]	
CR	1/30 (3.33%)
PR	8/30 (26.7%)
SD	14/30 (46.7%)
PD	7/30 (23.3%)
**Outcome of study completion**	
Dead	17/30 (56.7%)
Alive	13/30 (43.3%)

[1] CR: complete response, PR: partial response, SD: stable disease, PD: progression disease
